# Energy Expenditure in Critically Ill Obese Patients—A Prospective Observational Study

**DOI:** 10.3390/nu17132060

**Published:** 2025-06-20

**Authors:** Geraldine de Heer, Christoph Burdelski, Constantin Ammon, Anna Leonie Doliwa, Pascal Hilbert, Stefan Kluge, Jörn Grensemann

**Affiliations:** Department of Intensive Care Medicine, University Medical Center Hamburg-Eppendorf, Martinistraße 52, 20246 Hamburg, Germany

**Keywords:** nutrition obesity indirect calorimetry, energy expenditure

## Abstract

**Introduction:** In critically ill obese patients, both overfeeding and underfeeding have been associated with worsened outcomes, especially in mechanically ventilated patients. While indirect calorimetry (IC) is recommended to measure energy expenditure (EE), it is not widely available, and predictive formulas often lack accuracy. This study aimed to assess EE in critically ill obese patients and compare it with septic, non-obese patients as controls using IC. **Methods:** This prospective observational study was conducted at the University Medical Center Hamburg-Eppendorf, Germany, with 116 intensive care beds. EE was measured using IC at three timepoints: day 2–3 (acute phase), day 5–7 (end of acute phase), and day 10–15 (post-acute phase). Different reference weights were used to calculate EE, including ideal body weight (IBW), adjusted body weight (ABW), and total body weight (TBW). Nitrogen balance was also assessed to evaluate protein requirements. **Results:** We included 50 patients (28 obese and 22 controls). Equivalence between groups was found when ABW was calculated using 18% of excess body weight (EBW) at a mean of 24.4 kcal/kg/d for both groups. EE at the respective timepoints was 24.0 (95% confidence intervals: 22.1; 25.9), 24.2 (22.0; 26.5), 25.1 (21.4; 28.8) in obese and 24.9 (22.7; 27.0), 23.2 (20.7; 25.6), and 25.3 (21.8; 28.7) kcal/kg/d in control patients. Both groups exhibited a negative nitrogen balance, with the control group achieving nitrogen equilibrium by the post-acute phase. **Conclusions:** This study supports the ESPEN recommendation to base nutrition on ABW with 20% of EBW in obese critically ill patients when IC is unavailable. Further research is needed to determine optimal protein supplementation strategies and their timing to improve outcomes in this patient population.

## 1. Background

The incidence of obesity is a growing problem worldwide, and its prevalence is estimated at around 20% in the intensive care unit [1]. To appropriately feed these patients poses a challenge and both over- and underfeeding have been linked to decreases in favorable outcomes, especially in mechanically ventilated patients [2]. While indirect calorimetry has been recommended to measure energy expenditure (EE) for guidance of nutritional requirements, this method is not ubiquitously available [3,4], and predictive formulas show a high bias and low precision [5]; its use may also be associated with suboptimal outcome, although only few data are available, so far [6].

In patients with obesity, defined as a body mass index (BMI) above 30 kg/m^2^, metabolic processes differ, particularly in those with underlying insulin resistance. Proteolysis is accelerated and there is an altered mobilization of fuel stores [7]. To date, there is a paucity of data concerning feeding practices in critically ill obese patients and published studies include only small numbers of patients [8,9,10]. The current recommendation in these patients consists of a low caloric–high protein feeding formula [11,12] or the use of a pragmatic approach for the estimation of EE using a reference body weight calculated by adding 20 to 25% of the difference between total and ideal body weights to the ideal body weight if indirect calorimetry is not available [3]. However, this concept has not been proven in prospective evaluations; but, it could be helpful for the estimation of nutritional requirements in cases where indirect calorimetry is not available.

Therefore, we studied the EE measured by indirect calorimetry in critically ill patients with obesity in a prospective, observational study. Critically ill non- obese patients suffering from sepsis of any cause served as controls. We aimed to determine a reference weight (e.g., ABW) for the calculation of EE that would be suitable for both critically ill obese patients and critically ill non-obese patients.

## 2. Methods

### 2.1. Ethics

The study was approved by the Ethics Committee of the Hamburg Chamber of Physicians (2021-10519-BO-ff, approval: 18 June 2021, chairman Prof. Dr. Stahl). Due to the observational and non-interventional design, consent was waived. The study was conducted in accordance with the Declaration of Helsinki and adheres to the applicable STROBE guidelines.

### 2.2. Study Design and Setting

This prospective observational study was conducted at the Department of Intensive Care Medicine at the University Medical Center Hamburg-Eppendorf, Germany, with 12 intensive care units (ICU) with a total of 116 beds.

### 2.3. Participants

We included patients if they were at least 18 years old and had a body mass index of 30 kg·m^−2^ or above. Patients without obesity suffering from sepsis served as the control group. Sepsis was defined according to the SEPSIS-3 definition, with an increase in the Sequential Organ Failure Assessment (SOFA) score of 2 points or more [13].

Exclusion criteria were extracorporeal circulation (i.e., extracorporeal membrane oxygenation), renal replacement therapy (at the current ICU stay or before), air leaks from the respiratory system (i.e., chest tube with leakage), the requirement of an inspiratory fraction of oxygen above 0.7, supplemental oxygen therapy in awake patients, and co-medication with catechol-o-methyl-transferase inhibitors (i.e., entacapone, tolcapone), tricyclic antidepressants (TCA) or selective norepinephrine reuptake inhibitors (SNRI) as described before [14].

### 2.4. Nutritional Therapy

Nutritional therapy was conducted as recommended by the German guideline on nutritional therapy in critically ill patients: We commenced nutrition within 24 h after admission as trophic feeding and increased nutrition according to individual tolerance [4]. We targeted 75% of the measured EE until day 4 to 7, and increased to 100% nutritional support up to day 7. Parenteral nutrition was supplemented to achieve nutritional targets. For parenteral proteins, Aminoplasmal 10% (B. Braun, Melsungen, Germany) was used. We applied a correction factor of 1.2 to calculate the respective dose of amino acids [15]. In awake patients with adequate swallowing reflexes, oral nutrition was provided ad libitum, and the protein intake was estimated according to the recommendations on nutritional therapy in patient care [16].

### 2.5. Measurements

For indirect calorimetry, we used the Q-NRG + (COSMED, Rome, Italy) in either “canopy” or “vent-mode”, as appropriate. The system was set up according to the manufacturer’s specifications. Measurements were obtained at three timepoints: on day 2 or 3 (timepoint 1, “acute phase”), between day 5 and 7 (timepoint 2, “end of acute phase”), and optionally between day 10 and 15 after admission (timepoint 3, “post-acute phase”). Indirect calorimetry readings were averaged over at least 5 min as recommended by the manufacturer. Measurements were performed twice on the day of measurement and the mean values used for further calculations. Patients rested two hours before calorimetry.

Furthermore, 24 h urine nitrogen was measured, and the nitrogen balance was calculated as total protein intake/6.25 − (total urine nitrogen + 4 g) [17].

### 2.6. Outcome Parameters

The primary endpoint was the difference between mean EE per weight between the groups calculated for different reference weights: ideal body weight (IBW), adjusted body weight (ABW), and total body weight (TBW). ABW was calculated with different proportions of excess body weight (EBW) to find equivalence between the groups on EE per weight. Secondary endpoint was the nitrogen balance.

TBW was obtained from the patients’ records or, if unknown, measured with a bed scale (seca 985, seca, Hamburg, Germany). IBW was calculated as 91·(height [m] − 1.524) +50 for males or +45.5 for females [18]. EBW was calculated as TBW-IBW, and ABW as IBW + EBW × factor. As factors, we used 20% and 25% due to the recommendation of the ESPEN, and 40% due to its use in drug pharmacokinetics. Furthermore, we used further factors to find equivalence between the groups on EE per weight.

### 2.7. Sample Size

No formal sample size calculation was conducted; we aimed to include at least 20 patients per group.

### 2.8. Statistics

Statistical analyses were performed using SPSS (version 29, IBM Inc., Armonk, NY, USA) and Microsoft Excel 365 (Microsoft Corporation, Redmond, WA, USA). For the analyses, we used *t*-tests, Chi-square and Fisher’s tests, and generalized mixed models, as applicable. Two-tailed *p*-values <0.05 were regarded as statistically significant.

## 3. Results

From 1 July 2021 to 28 February 2023, we included a total of 50 patients with 28 patients with obesity and 22 controls. An overview of patients’ characteristics is depicted in Table 1.

**Figure 1:**
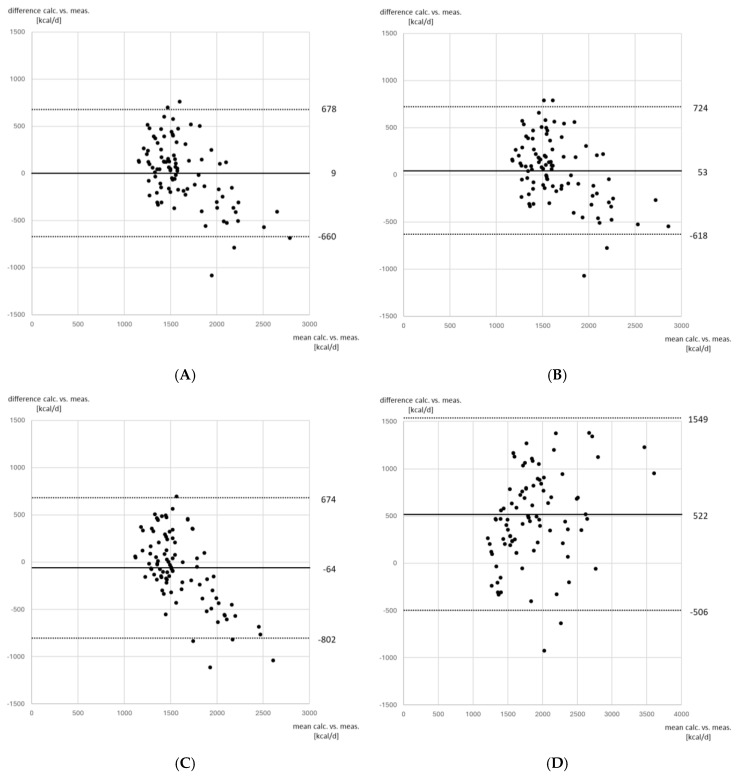
Bland−Altman plots for agreement between indirect calorimetry and calculation of energy expenditure using different reference weights. (**A**) Indirect calorimetry versus adjusted body weight calculated using 18% of excess body weight; (**B**) indirect calorimetry versus adjusted body weight calculated using 25% of excess body weight; (**C**) indirect calorimetry versus ideal body weight; (**D**) indirect calorimetry versus total body weight. For the calculation of energy expenditure by reference weight, 24 kcal/kg/d were used. The solid line depicts bias, the dotted lines depict the limits of agreement as 1.96 × standard deviation of the difference between both methods. Bias and limits of agreement are given as kcal/d.

An overview of mean energy expenditure is given in Table 2a,b. The generalized linear mixed model analysis for groups and timepoints revealed significant differences between groups at timepoint 1 for mean EE based on ABW calculated with 40% of EBW and for TBW, at timepoint 2 for EE and for mean EE based on IBW, and for mean EE based on TBW for all timepoints (Table 2a,b). Equivalence between groups on EE per weight was found at an ABW using 18% of EBW for calculation with a mean EE of 24.4 (95% confidence intervals: 23.0; 25.9) kcal/kg/d for both groups. Bland–Altman plots are shown in Figure 1A–D for the agreement between indirect calorimetry and the estimation of EE by the calculation of 24 kcal/kg/d with reference weights of ABW using 18% and 25% of EBW, IBW, and TBW. Concerning nitrogen balance, both groups exhibited a negative nitrogen balance with a nadir at timepoint 2. In the control group, nitrogen balance significantly increased thereafter reaching nitrogen equilibrium at timepoint 3 (Table 3).

## 4. Discussion

We measured the EE of critically ill obese patients compared to a control group of patients suffering from sepsis. We calculated different reference weights and found equivalence between the groups for an ABW with 18% of EBW.

Adequate nutrition in critically ill obese patients has been a matter of debate, and nutrition is an important factor influencing patients’ outcome with data indicating that both under- and overfeeding leads to an increase in mortality [19]. In patients with obesity, an estimation based to TBW would certainly lead to overfeeding because the base metabolic rate of adipose tissue at 4.5 kcal/kg/d is far less than that of muscle tissue at 13 kcal/kg/d [20]. Furthermore, obese patients may differ in their body composition with some patients being sarcopenic while others retain a physiological muscular mass [21].

Ideally, indirect calorimetry should be used and prescription of nutrition should be based on the measured EE. Unfortunately, indirect calorimetry is not ubiquitously available and many clinicians rely on predictive formulas when prescribing nutrition to their patients [22], which show a low precision, particularly in patients with obesity [23].

Current guidelines, despite being based on the same data, reach different recommendations on feeding of obese patients. In the absence of indirect calorimetry, guidelines as, e.g., the American Society for Parenteral and Enteral Nutrition (ASPEN) recommend 11–14 kcal/kg/d of TBW for patients with BMI between 30 and 50 kg/m^2^ and 22–25 kcal/kg/d of IBW for patients above a BMI of 50 kg/m^2^ [12]. This concept was derived from a single study categorizing patients in the respective BMI ranges and evaluating different reference weight concepts [24]. This concept has also been adopted by the German guideline for clinical nutrition in critical care medicine [4]. However, this concept could prove troublesome in clinical practice. A hypothetical patient with a BMI of 50 and a height of 1.80 m has a mass of 162 kg. According to the ASPEN and the German recommendation, this patient would receive 2025 kcal/d (12.5 kcal/kg/d × 162 kg TBW). Any increase in the patients’ mass would categorize this patient in the super-obese category (BMI above 50 kg/m^2^), leading to a suggested prescription of 1680 kcal/d (23.5 kcal/kg/d * 71.5 kg IBW), irrespective of actual weight. In our opinion, this concept, in which the required nutrition is lower in higher body weights should be viewed critically and does not take into account the metabolic base rate of adipose tissue, as outlined above [20]. On the other hand, the ESPEN suggests a pragmatic approach basing nutrition on ABW with 20 to 25% of EBW [3]. This approach better reflects the metabolic rate of adipose tissue at approximately 1/3 of the respective rate of muscle tissue [20] but lacks validation, so far. In our study, we show that the EE/kg of obese patients was best reflected when the reference weight was calculated as ABW with 18% of EBW, thus supporting the ESPEN recommendation of ABW calculated with 20 to 25% of EBW. Further studies are required to allow for the generalization of our results, although our findings are supported by physiological data, as outlined above.

Considering the provision of proteins, a high-protein–low-calorie diet has been evaluated in non−randomized trials and has been adopted by the ASPEN guideline recommendation suggesting supplementation of proteins in a range from 2.0 g/kg/d IBW for patients with a BMI of 30–40 kg/m^2^ and up to 2.5 g/kg/d IBW for patients with a BMI ≥ 40 kg/m^2^. In contrast the ESPEN and the German guidelines suggests fewer proteins. The ESPEN guideline recommends 1.3 g/kg/d of ABW above a BMI of 30 kg/m^2^, and the German guideline recommends 1.5 g/kd/d of IBW above a BMI of 30 kg/m^2^ [3,4], if no calorimetry and nitrogen balance are available. Interestingly, the protein provision is fixed above certain BMI thresholds in the ASPEN and German guidelines, because it solely relies on the IBW that is calculated only from patients’ height. To date, no data are available on the protein requirements of adipose tissue and further studies are urgently required [25]. The concept of high-protein delivery has been recently challenged by a meta-analysis using a Bayesian approach, showing that high protein delivery is presumably associated with harm and a reduced quality of life after ICU discharge [26]. A post hoc trial of a randomized controlled trial showed no benefit of high protein supplementation in critically ill obese patients [27]. Furthermore, different approaches on determining the reference weight for protein supplementation have been suggested [28]. Protein requirements mainly depend on the lean body mass that may be difficult to determine under clinical conditions. Without measurements of body composition and thus lean body mass, it may not be possible to distinguish between obese sarcopenic and obese non-sarcopenic patients, rendering it virtually impossible to deliver a targeted protein supplementation.

Another method of guiding protein supplementation is to adapt protein delivery according to nitrogen balance, aiming for nitrogen equilibrium. It must be noted that, particularly in the acute phase of any critical illness, catabolism prevails, and that nitrogen equilibrium may only be achieved after the acute phase. On the other hand, data indicates that nitrogen equilibrium may be reached earlier by increasing protein supply which was associated with a benefit on outcome in some patient populations [29,30]. In our study, we found no significant differences between groups, but nitrogen equilibrium was only reached in the control group after day 7, after the acute phase should have ended. We must admit that protein delivery in our study was below the current guideline recommendations which we attribute to differences in prescribed and delivered nutrition, which unfortunately poses a common problem in many ICUs that needs to be addressed for the optimization of nutritional therapy [31,32,33]. In clinical practice, it is often unknown if catabolism is caused by the acute phase of critical illness or by insufficient protein delivery. Thus, it may be tempting to supply high protein doses early in the course of illness to shorten patients’ time in a catabolic state, but it has been shown that high protein doses may be harmful, particularly in patients with organ failure [34] and a post hoc analysis of this study including obese patients did not find a benefit for the high protein group either [27]. Therefore, further studies should not only focus on the optimal protein quantities but also on determining the optimal timing in the course of critical illness when increasing protein delivery may safely be considered.

Our control group consisted of septic patients, because these patients present a common group of critically ill patients. During sepsis, pro-inflammatory cytokines and increased catecholamine concentrations increase EE [35]. However, the overall EE is reduced in the acute phase of critical illness [36] and hypermetabolism typically occurs in the later phase of disease [37,38]. Because our group of patients with obesity were also critically ill, the lean body mass metabolism should be similar in both groups and the excess EE in the obese group might be attributed to the adipose tissue. However, we did not perform measurements on body composition to determine lean body mass, particularly in the obese group.

Our study has the following further limitations. We chose to calculate the IBW according to the formula suggested by Devine [18]. Other suggested formulas, e.g., that by Peterson et al. [39] or the formula given in the ESPEN guideline [3], yield far lower values, which we deem inappropriately low, particularly regarding the latter formula. The protein delivery was below the current guideline recommendations which weakens our results, particularly concerning the nitrogen balance. Furthermore, several data were missing for timepoint 3, but this did not influence our primary analysis.

## 5. Conclusions

In our prospective observational study evaluating energy expenditure in critically ill obese patients compared to septic patients, we were able to confirm the ESPEN recommendations to provide nutrition based on ABW using 20% of EBW for calculations when indirect calorimetry is not available. Regarding protein requirements, further research is needed to determine the optimal timing and required quantities.

## Figures and Tables

**Table 1:** Patient characteristics.

Parameter	Obese (*n* = 28)	Control (*n* = 22)	*p*
Age (years)	62 ± 11	64 ± 16	0.23
Sex male	*n* = 15 (54%)	*n* = 11 (50%)	0.80
Height (cm)	172 ± 10	172 ± 9	0.48
BMI (kg/m^2^)	36.1 ± 4.9	23.8 ± 3.9	<0.001
IBW (kg)	65.9 ± 11.2	65.7 ± 9.2	0.44
ABW20 (kg)	74.2 ± 11.8	64.0 ± 10.4	<0.001
ABW40 (kg)	82.4 ± 13.2	65.7 ± 11.1	<0.001
TBW (kg)	107.3 ± 20.0	70.5 ± 14.1	<0.001
APACHE II	46 ± 11	51 ± 11	0.001
SAPS II	38 ± 12	43 ± 10	0.004
SOFA (day 1)	9 ± 3	10 ± 4	0.17

BMI: body mass index; IBW: ideal body weight; ABW20, ABW40: adjusted body weight calculated as ideal body weight plus 20% or 40% of excess body weight, respectively; TBW: total body weight; APACHE: Acute Physiology And Chronic Health Evaluation, SAPS: Simplified Acute Physiology Score, SOFA: sequential organ failure assessment; data are given as mean ± standard deviation or numbers and percentages, as applicable.

**Table 2:** (**a**) Mean energy expenditure at measured timepoints. (**b**) Mean energy expenditure per weight at measured timepoints.

(a)			
Group	Timepoint 1 (Day 2–3)	Timepoint 2 (Day 5–7)	Timepoint 3 (Day 12–15)
Obese	1767 (1593; 1941)	1812 (1617; 2007) *	1876 (1600; 2152)
Control	1575 (1379; 1771)	1463 (1250; 1676)	1608 (1338; 1877)
**(b)**			
**Group**	**Timepoint 1 (Day 2–3)**	**Timepoint 2 (Day 5–7)**	**Timepoint 3 (Day 12–15)**
IBW:			
Obese	26.7 (24.7; 28.8)	27.1 (24.7; 29.6) *	28.2 (24.3; 32.2)
Control	24.1 (21.8; 26.5)	22.3 (19.7; 25.0)	24.8 (21.1; 28.4)
ABW18:			
Obese	24.0 (22.1; 25.9)	24.2 (22.0; 26.5)	25.1 (21.4; 28.8)
Control	24.9 (22.7; 27.0)	23.2 (20.7; 25.6)	25.3 (21.8; 28.7)
ABW20:			
Obese	23.7 (21.8; 25.6)	24.0 (21.7; 26.2)	24.8 (21.1; 28.5)
Control	24.8 (22.7; 26.9)	23.1 (20.7; 25.5)	24.8 (21.1; 28.5)
ABW25:			
Obese	23.1 (21.2; 24.9)	23.3 (21.1; 25.5)	24.1 (20.5; 27.8)
Control	24.7 (22.6; 26.7)	23.0 (20.6; 25.4)	25.0 (21.6; 28.5)
ABW40:			
Obese	21.3 (19.5; 23.1) ^#^	21.5 (19.4; 23.7)	22.3 (18.7; 25.8)
Control	24.2 (22.2; 26.3)	22.6 (20.2; 24.9)	24.6 (21.2; 27.9)
TBW:			
Obese	16.5 (14.8; 18.3) ^$^	16.7 (14.6; 18.7) *	17.2 (13.9; 20.5) ^#^
Control	22.8 (20.8; 24.7)	21.3 (19.0; 23.5)	23.7 (20.0; 26.3)

For subtable (a): energy expenditure is given in kcal/d (mean and 95% confidence intervals in brackets); * *p* < 0.05 vs. control. For subtable (b): energy expenditure is given in kcal/kg/d (mean and 95% confidence intervals in brackets); IBW: ideal body weight; ABW: adjusted body weight, number after ABW refers to percentage of excess body weight used for calculation; * *p* < 0.01 vs. control; ^#^ *p* < 0.05 vs. control; ^$^ *p* < 0.001 vs. control.

**Table 3:** Nitrogen Balance at measured timepoints.

Group	Timepoint 1 (Day 2–3)	Timepoint 2 (Day 5–7)	Timepoint 3 (Day 12–15)
Obese	−4.4 (−8.3; −0.5)	−7.5 (−12.3; −2.8)	−3.5 (−10.1; 3.2)
Control	−5.5 (−9.9; −1.1)	−7.2 (−12.0; −2.2)	0.3 (−6.4; 7.1) *

Nitrogen balance is given in g/d (mean and 95% confidence intervals in brackets); * *p* < 0.05 vs. timepoint 2.

## Data Availability

The data are available from the corresponding author upon reasonable request. The data are not publicly available due to privacy reasons.
